# Haspin kinase inhibition dampens pseudorabies virus infection *in vitro*

**DOI:** 10.3389/fvets.2025.1572729

**Published:** 2025-04-23

**Authors:** Lei Tan, Yong Yang, Xiaojiu Huang, Youqing Yuan, Kaixin Wang, Xiaoye Peng, Yiyan He, Yijin Wang, Lei Lei, Yingyi Chen, Deyong Duan, Naidong Wang, Yi Yang, Feiyan Dai, Cuiqing Huang, Aibing Wang

**Affiliations:** ^1^College of Animal Science and Technology, Yangtze University, Jingzhou, China; ^2^Fujian Provincial Key Laboratory for Prevention and Control of Animal Infectious Diseases and Biotechnology, Longyan University, Longyan, China; ^3^College of Veterinary Medicine, Hunan Agricultural University, Changsha, China; ^4^Yunnan Sino-Science Gene Technology Co., Ltd., Kunming, China; ^5^Department of Chemistry, University College London, London, United Kingdom; ^6^College of Veterinary Medicine, Yunnan Agricultural University, Kunming, China

**Keywords:** pseudorabies virus, Haspin kinase, antiviral activity, CHR-6494, replication stage

## Abstract

Pseudorabies virus (PRV) represents a considerable infectious threat to the swine industry in China and poses potential health risks to humans. However, there is a notable lack of specific antiviral agents aimed at combating PRV. Haspin is involved in histone phosphorylation during mitosis, while the role of swine Haspin in PRV infection has not been previously investigated. In the present study, we demonstrated that Haspin expression was significantly enhanced in response to PRV infection. Overexpression of the *haspin* gene notably enhanced PRV infection, while genetic inhibition of *haspin* gene resulted in a substantial reduction in viral infection. Further investigations indicated that the Haspin kinase inhibitor CHR-6494 effectively suppressed PRV infection in a concentration-dependent manner, primarily by inhibiting viral virus replication rather than interfering with the processes of binding, entry, or release. Additionally, treatment with CHR-6494 effectively restricted Herpes simplex virus type 1 infection in Vero cells. Collectively, these findings indicate that Haspin may serve as a novel therapeutic target for the management of infections caused by *Alphaherpesvirinae*.

## 1 Introduction

Pseudorabies (PR) is an acute infectious disease caused by pseudorabies virus (PRV), which is primarily characterized by fatal encephalitis and neurological disorders in piglets, as well as reproductive failures in sows (1). Pigs are recognized as the principal reservoir for PRV, while this virus is capable of infecting a variety of animal species (2). Moreover, there is compelling evidence indicating that PRV can also infect humans, as demonstrated by the isolation of a variant strain from a patient diagnosed with acute encephalitis in China (3).

Based on the genetic characteristics of PRV strains, these strains have been classified into two distinct genotypes, namely genotype I and genotype II (2). Predominantly, the PRV strains present within the Chinese swine industry are classified under genotype II, which can be further subdivided into two groups: variant PRV strains and classical PRV strains. It is noteworthy that classical PRV strains were first identified in the 1990s, whereas variant PRV strains emerged subsequent to 2011 (2). Currently, the majority of PRV strains circulating in China are identified as variant PRV strains (4–6).

Haploid germ cell-specific nuclear protein kinase (Haspin) is a nuclear serine–threonine kinase that plays a crucial role in the regulation of normal mitosis in mammalian cells (7). Although Haspin is typically expressed at low levels in proliferating normal somatic cells, its expression is markedly elevated in a variety of human cancers, including pancreatic and colorectal malignancies (7, 8). Moreover, the inhibition or knockdown of Haspin effectively reduced the proliferation of multiple cancer cell types, indicating that Haspin may serve as a viable therapeutic target for cancer treatment (7–9). However, the role of Haspin in viral infections remains to be explored.

In the present study, we investigated the role of Haspin in PRV infection and further analyzed the antiviral efficacy of the Haspin inhibitor CHR-6494 against PRV infection *in vitro*. Our findings indicated that PRV infection led to a significant upregulation of Haspin expression. Overexpression of the *haspin* gene promoted PRV infection, while genetic inhibition of *haspin* gene significantly inhibited viral infection. CHR-6494 efficiently suppressed PRV infection across different cell lines, with its inhibitory effects mainly occurring during the stage of viral replication phase, rather than at the stage of binding, entry, or release. Additionally, CHR-6494 was shown to inhibit Herpes simplex virus (HSV-1) infection *in vitro*.

## 2 Materials and methods

### 2.1 Cells, viruses, and antibodies

PK15, Vero E6, and 3D4/21 cell lines were maintained in our laboratory and cultured in DMEM supplemented with 10% fetal bovine serum (FBS) and 1% penicillin–streptomycin (P/S) at 37°C with 5% CO_2._ Various strains of PRV, including a variant strain, a classical strain, and a recombinant PRV strain expressing EGFP, as well as strains of Herpes simplex virus type 1, were preserved in our laboratory. Antibodies targeting Haspin (YN0132) and *β*-actin (YM8343) were purchased from Immunoway (USA), the primary mouse monoclonal antibodies against PRV gB were generously provided by Professor Ping Jiang from Nanjing Agricultural University, China.

### 2.2 Cytotoxicity assay

Cells were cultured in 96-well plates and incubated with DMEM medium supplemented with 10% FBS, along with different concentrations of the Haspin inhibitor (CHR-6494) or DMSO for 36 h. The assessment of cell viability was conducted using the MTT assay, as described in the study by Xiong et al. (10).

### 2.3 Western blot analysis

Total proteins were extracted using NP40 lysis buffer containing protease inhibitors. Equal amounts of protein samples were separated by 10% SDS-PAGE and transferred onto PDVF membranes. The membranes were blocked with 3% bovine serum albumin for 1 h, and incubated with specific primary antibodies overnight at 4°C. Finally, the membranes were treated with the secondary antibodies for 1 h. The target proteins on the membranes were visualized using an enhanced chemiluminescence substrate (ECL) kit.

### 2.4 RT-qPCR

Total RNA was extracted from the cells utilizing TRIzol reagent (TIANGEN, China). Approximately 1 μg of RNA was then employed for cDNA synthesis using a commercial reverse transcription kit (Promega, United States). The RT-qPCR assay was conducted with SYBR Fast qPCR kits (Vazyme, China) and specific primers (Supplementary Table 1).

### 2.5 Plasmids, shRNA, and transfection

The complete coding sequence of the porcine Haspin gene was amplified by RT-PCR and cloned into the pCI-NEO vector to generate the pCI-Haspin plasmid. Two shRNA sequences targeting the porcine Haspin gene were individually inserted into the pLKO.1-EGFP-Puro vector. Transfection procedures were conducted using Lipofectamine 2000 (Invitrogen, United States) according to the manufacturer’s instructions.

### 2.6 Statistical analysis

All experiments conducted in this study were executed in triplicate or more. Data from different groups were analyzed using a *t*-test with GraphPad Prism Version 8.0 (GraphPad Software, La Jolla, CA, United States). Statistical significance was shown by * *p* < 0.05, ** *p* < 0.01, *** *p* < 0.001.

## 3 Results

### 3.1 Haspin was involved in PRV infection

**Figure 1:**
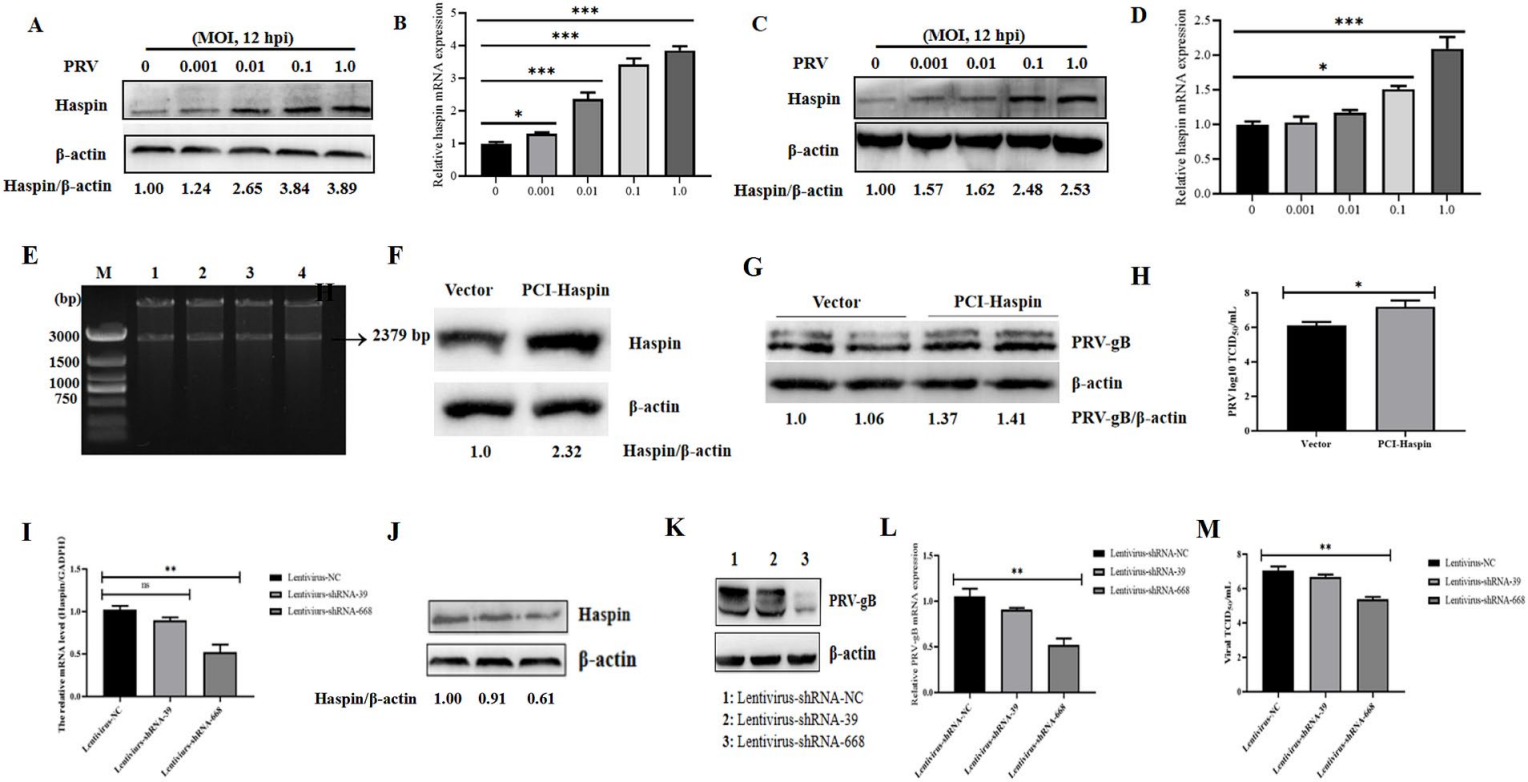
Haspin is involved in PRV infection. **(A,C)** 3D4/21 and PK15 cells were infected with PRV-WH for 12 h at different MOIs of 0.001, 0.01, 0.1 and 1.0, after which, the Haspin protein levels were assessed using western blot. **(B,D)** 3D4/21 and PK15 cells were infected with PRV-WH for 12 h at different MOIs of 0.001, 0.01, 0.1 and 1.0, RT-qPCR assay was conducted to analyze Haspin mRNA level. **(E)** Enzyme digestion validation of pCI-Haspin recombinant vector. **(F)** 3D4/21 cells were transfected with either an empty vector (pCI-NEO, 2 μg) or the pCI-Haspin plasmid (2 μg) for 36 h, the cells were then harvested to assess the Haspin expression, with *β*-Actin serving as the internal control. **(G,H)** The empty vector (pCI-NEO, 2 μg) or pCI-haspin plasmid (2 μg) were transfected into 3D4/21 cells for 36 h, followed by infection with PRV-WH at an MOI of 0.1. Both cells and supernatants were collected to evaluate the relative expression of PRV-gB and to determine viral titers, respectively. **(I,J)** The levels of Haspin mRNA and protein in 3D4/21 cells transfected with lentivirus-NC, lentivirus-shRNA-39, or lentivirus-shRNA-668 plasmids, were analyzed using RT-qPCR and western blot techniques, respectively. **(K–M)** The lentivirus-NC, lentivirus-shRNA-39, or lentivirus-shRNA-668 plasmids were transfected into the 3D4/21 cells for 36 h, followed by infection with PRV-WH (MOI = 0.1). Cells and supernatants were collected to assess PRV-gB protein expression, mRNA levels, and viral titers.

We first assessed the relative expression levels of Haspin in PRV-infected 3D4/21 and PK15 cells at 12 h post-infection (hpi). The findings revealed a significant increase in both mRNA and protein levels of Haspin in these cell lines following PRV infection (Figures 1A–D). Next, we explored the impact of Haspin overexpression and knockdown on PRV infection in 3D4/21 cells. The results demonstrated that both the protein expression levels and viral titers were considerable higher in the Flag-Haspin group (Figures 1E–H). Conversely, the knockdown of haspin gene in 3D4/21 cells resulted in the reduction in both protein and mRNA expression levels, as well as a decrease in viral titers (Figures 1I–M). Collectively, these findings indicate that porcine Haspin is involved in PRV infection.

### 3.2 CHR-6494 exhibits antiviral activity against PRV infection

**Figure 2:**
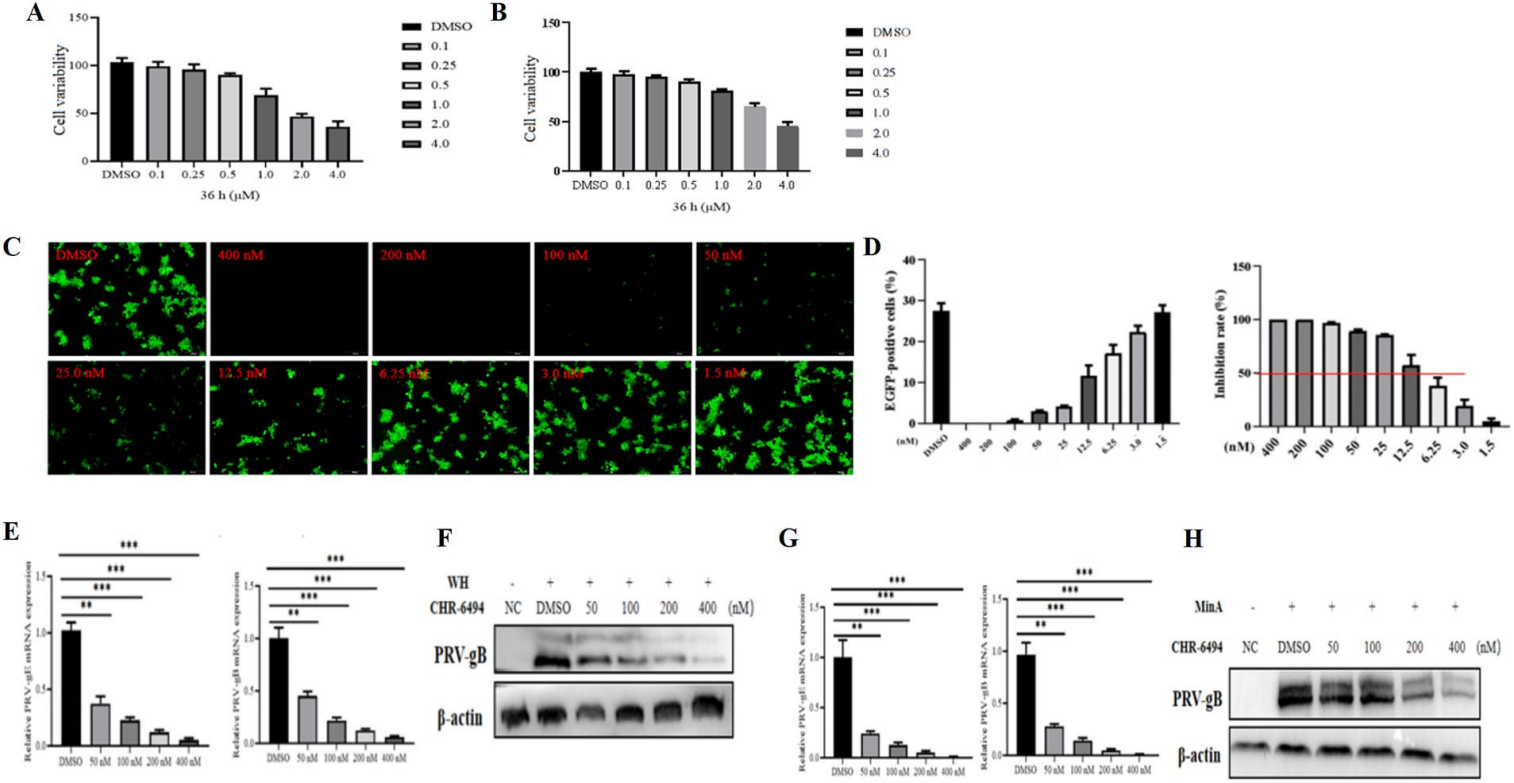
CHR-6494 demonstrates antiviral properties against PRV infection. **(A,B)** PK15 and 3D4/21 cells were treated with CHR-6494 at concentrations ranging from 0 to 4 μM for 36 h. After which, cell viability was assessed via the MTT assay (%). **(C)** 3D4/21 cells were treated with CHR-6494 at concentrations between 0 and 400 nM for 2 h and then infected with rPRVHuN-EGFP (10^3^ TCID_50_) for 24 h. The expression of EGFP in 3D4/21 cells was visualized using an Olympus microscope (Scale bar = 100 μm). **(D)** The proportion of EGFP-positive 3D4/21 cells was quantified using the Image J software. **(E–H)** 3D4/21 cells were pre-treated with CHR-6494 (50, 100, 200, or 400 nM) for 2 h, and then infected with either the PRV-WH or PRV-MinA strain for 24 h. RT-qPCR and western blot analyses were performed to evaluate the mRNA levels of PRV gB and gE genes **(E,G)** and the protein expression of PRV gB **(F,H)**.

To investigate the impact of Haspin on PRV infection, we administered CHR-6494, a selective inhibitor of Haspin kinase that is known to influence the phosphorylation of histone H3T3 (4), to 3D4/21 and PK15 cell lines. The cytotoxicity of CHR-6494 on 3D4/21 and PK15 cell lines was assessed through MTT assays. The results demonstrated that the maximum non-cytotoxic concentration of CHR-6494 for both PK15 and 3D4/21 cells was approximately 500 nM (Figures 2A,B).

The antiviral efficacy of CHR-6494 was evaluated using a recombinant PRV (rPRVHuN-EGFP) that expresses EGFP. 3D4/21 cells were pre-treated with various concentrations of CHR-6494 for 2 h, followed by infection with rPRVHuN-EGFP (1,000 TCID_50_) for 24 h. The proportion of EGFP-positive cells in each group was subsequently quantified. The results revealed that CHR-6494 treatment significantly decreased the percentage of EGFP-positive cells in a dose-dependent manner (Figures 2C,D). Based on these observations, the half-maximal inhibitory concentration (IC_50_) of CHR-6494 against rPRVHuN-EGFP infection was estimated to be 8.35 nM, indicating a selectivity index (SI) of 59.88.

To further investigate the anti-PRV efficacy of CHR-6494, both classical and variant PRV strains were selected for examination. 3D4/21 cells were pre-treated with CHR-6494 for 2 h, after which they were infected with either classical or variant PRV strains. At 24 hpi, the cells were washed with PBS and subsequently harvested to evaluate viral replication efficiency through western blot and RT-qPCR assays. The results demonstrated that treatment with CHR-6494 significantly decreased the mRNA levels of PRV gE and gB for both variant and classical strains in a dose-dependent manner (Figures 2E,G). Furthermore, CHR-6494 significantly inhibited the synthesis of PRV gB protein (Figures 2F,H). Collectively, these findings suggest that CHR-6494 effectively inhibits the infections caused by both classical and variant PRV strains *in vitro*.

### 3.3 CHR-6494 inhibits PRV replication rather than entry and attachment

**Figure 3:**
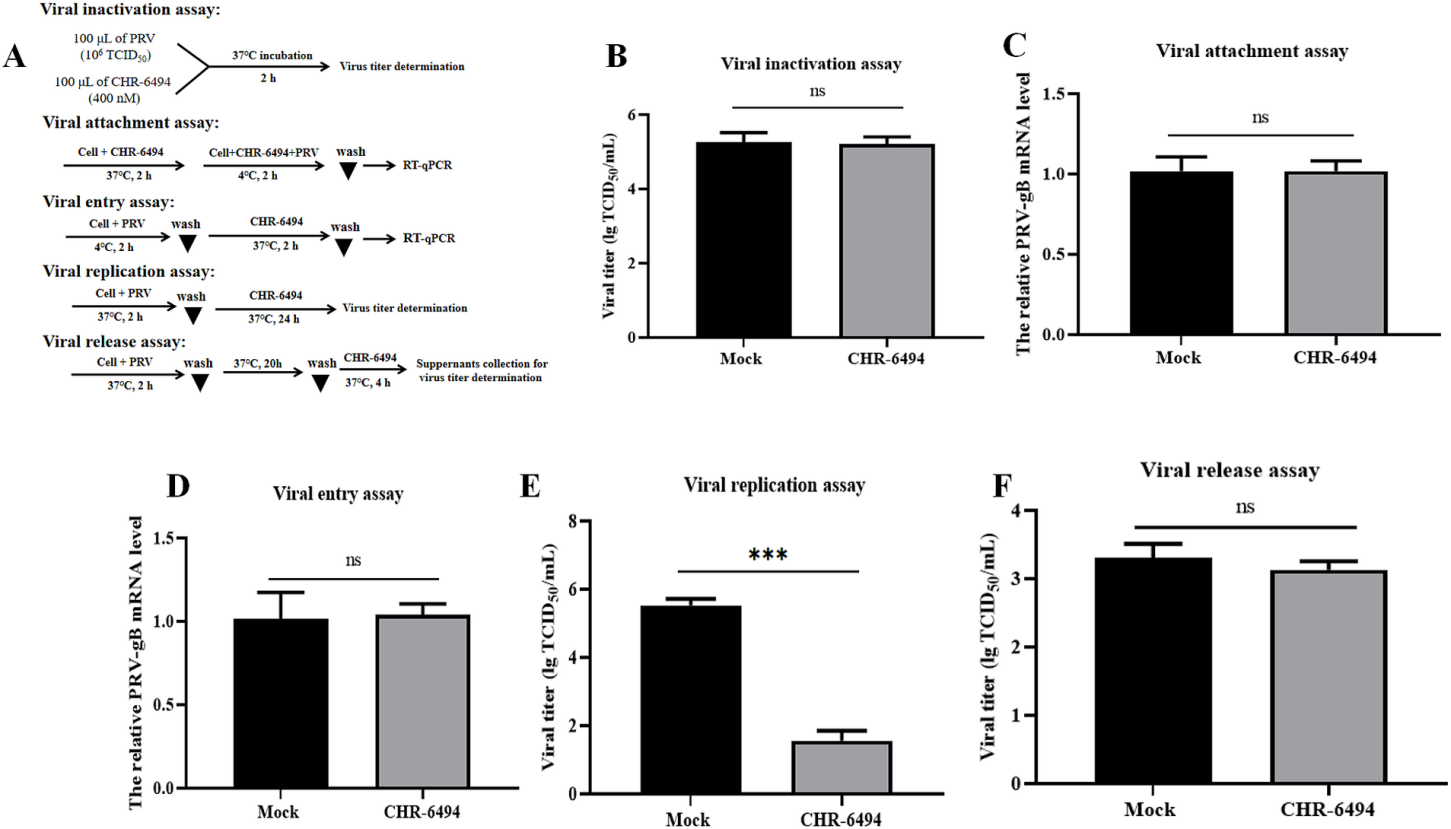
Effects of CHR-6494 treatment on different stages of PRV infection. **(A)** A schematic representation of various treatment conditions. **(B)** Virus inactivation assay. **(C)** Virus attachment assay. **(D)** Virus entry assay. **(E)** Virus replication assay. **(F)** Virus release assay.

Utilizing the PRV-WH strain as a model, we examined the impact of CHR-6494 treatment on various phases of the PRV life cycle, including viral attachment, entry, replication, and release. Furthermore, we assessed the potential of this compound to directly inactivate the virus (Figure 3A). The results indicated that CHR-6494 treatment led to a significant reduction in viral titers during the replication stage (Figure 3E). Conversely, no notable differences in viral titers were detected between the CHR-6494-treated and control groups in the assays assessing virus inactivation and release (Figures 3B,F). Additionally, the relative expression levels of PRV-gB mRNA did not exhibit significant differences between the experimental and control groups in the assays for viral attachment and entry assays (Figures 3C,D). Taken together, these findings imply that Haspin is involved in PRV replication but not in the attachment, entry, or release stages of the viral life cycle.

### 3.4 CHR-6494 treatment effectively inhibited PRV genes and PRV-induced cellular gene transcription

To investigate the impact of CHR-6494 treatment on the transcription of PRV genes and PRV-induced cellular genes induced by PRV, 3D4/21 cells were pre-treated with CHR-6494 (200 nM) and then infected with PRV (10^3^ TCID_50_). Total RNA was extracted at 12 hpi and 24 hpi, and analyzed using RT-qPCR assay to quantify the mRNA expression levels of various PRV genes, including the immediate-early gene (IE180), early genes (EPO and US1) and other replication-associated genes (UL42, UL30, and UL9). Additionally, the expression levels of inflammation-related genes (TNF-*α*, IL-6, and IL-1) and innate immunity-related genes (IFN-*β*, IFITM1, and IFITM3) were assessed. The results indicated that CHR-6494 significantly reduced the mRNA expression levels of several viral genes (Supplementary Figure 1A). Furthermore, PRV infection resulted in an elevation of mRNA expression levels of TNF-α, IL-6, and IL-1, which contributed to a cellular cytokine storm; however, CHR-6494 treatment effectively mitigated this PRV-induced cytokine storm (Supplementary Figure 1B). Similarly, PRV infection caused a downregulation of innate immunity-related genes, which was reversed by CHR-6494 treatment (Supplementary Figure 1C).

### 3.5 CHR-6494 treatment inhibited HSV-1 infection *in vitro*

**Figure 4:**
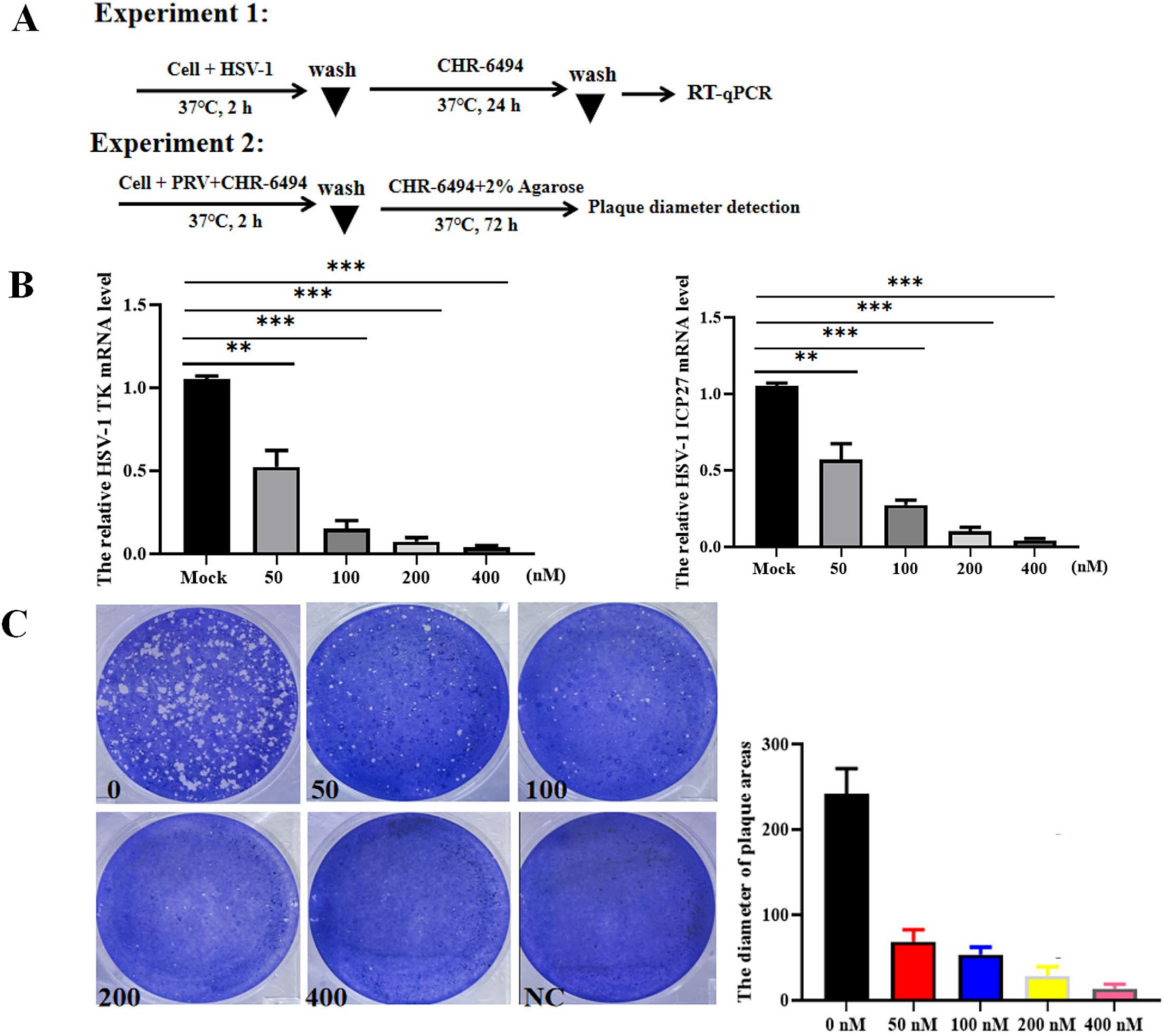
CHR-6494 inhibits HSV-1 infection *in vitro.* **(A)** A schematic overview of different treatment conditions. **(B)** Vero cells were pre-treated with CHR-6494 (50, 100, 200, or 400 nM) for 2 h, and then infected with HSV-1 strain with an MOI of 0.01 for 24 h. RT-qPCR assay was performed to analyze the mRNA levels of HSV-1 TK and ICP27 genes. **(C)** Vero cells were treated with CHR-6494 (50, 100, 200, or 400 nM) and then infected with 10^3^ TCID_50_ of HSV-1 for 72 h. The diameters of the plaques resulting from HSV-1 infection in different treatment groups were measured.

To explore the broad-spectrum antiviral efficacy of CHR-6494 against other *Herpesvirus* infections, its impacts on HSV-1 infection were examined. Vero cells were infected with HSV-1 (MOI = 0.01) for 2 h, followed by incubation with CHR-6494 (200 nM). At 24 hpi, total RNA was extracted from the cells and subjected to RT-qPCR to quantify the mRNA levels of HSV-1 genes ICP27 and TK (Figure 4A). In a separate experiment, Vero cells were exposed to CHR-6494 and then infected with 10^3^ TCID_50_ of HSV-1 for 2 h at 37°C to facilitate viral entry. The culture medium was then replaced with DMEM containing 2% FBS, 2% agarose, and CHR-6494 at 200 nM. At 72 hpi, the diameters of the plaques in each experimental group were measured (Figure 4A). The results demonstrated that CHR-6494 effectively inhibited HSV-1 replication *in vitro*, as evidenced by a marked reduction in the mRNA levels of HSV-1 TK and ICP27 following treatment with CHR-6494 (Figure 4B). Furthermore, the plaques resulting from HSV-1 infection in Vero cells treated with CHR-6494 exhibited significantly smaller diameters compared to those in the control group, with this effect being dose-dependent (Figure 4C).

## 4 Discussion

Epigenetics is defined as the heritable changes in gene expression or function that lead to phenotypic alterations, without altering the fundamental DNA sequence. Histones, which serve as crucial structural proteins within chromosomes, are classified into five subtypes Histone 1 (H1), H2A, H2B, H3, and H4. Histone acetylation, phosphorylation, and methylation are key epigenetic modifications within the mammalian genome. Notably, the inhibition of histone deacetylase 1 has been demonstrated to effectively suppress infections caused by various viruses, including PRV (11), HSV-1 (12), and Human immunodeficiency virus (13). Conversely, the activation of histone deacetylase 1 has been found to enhance PEDV replication in porcine IPEC-J2 cells (14), highlighting the significant role of histone acetylation in viral infections. Haspin kinase, which is implicated in histone phosphorylation, plays a significant role in the regulation of cell cycle and differentiation processes in mammalian cells.

In this study, we examined the role of Haspin kinase in PRV infection for the first time. Our findings revealed a significant upregulation of both of the mRNA and protein levels of Haspin in PK15 and 3D4/21 cell lines following PRV infection, suggesting a potential involvement of Haspin in the viral life cycle. We subsequently assessed the functions of Haspin in PRV infection by comparing the replication characteristics of PRV in 3D4/21 cells with *haspin* overexpression, knock-down, and wild-type conditions. The data demonstrated that overexpression of *haspin* gene enhanced PRV replication efficiency, whereas knockdown of *haspin* gene inhibited resulted in a reduction of viral replication.

CHR-6494, the first-in-class agent known to inhibit Haspin kinase by reducing the phosphorylation of histone H3 at Thr3 residue, has shown effective anti-cancer and anti-tumor activities (15, 16). We therefore investigated the impact of inhibiting Haspin kinase activity using CHR-6494 in PRV infection. Our findings indicated that CHR-6494 inhibited PRV infection in 3D4/21 cells in a dose-dependent manner. Specifically, the proportion of EGFP-positive 3D4/21 cells infected with the PRVHuN-EGFP strain, as well as the mRNA levels of PRV gE and gB in wild-type PRV-infected cells treated with CHR-6494, were significantly lower compared to the control group. Further analysis revealed that CHR-6494 treatment inhibited PRV infection by suppressing virus replication, without affecting the processes of viral attachment, entry or release steps. In addition, we observed that CHR-6494 also inhibited HSV-1 replication *in vitro*. These findings align with the critical role of Haspin kinase in mammalian cell mitosis, which is intricately linked to the replication of DNA viruses, including Human papillomavirus (17) and Epstein–Barr virus (18).

Immediate-early genes, early genes, and other essential genes are integral to the replication of PRV and the generation of progeny virus (19). This study examined the impacts of CHR-6494 on the expression of PRV genes, specifically targeting the immediate-early gene (IE180), early genes (EPO and US1), and other replication-essential genes (UL42, UL30, and US9). The findings demonstrated that CHR-6494 significantly inhibited the mRNA levels of all PRV genes examined, suggesting that this compound may suppress PRV replication by obstructing the transcription or expression of these genes. Additionally, this study revealed that PRV infection led to an upregulation of mRNA levels associated with inflammatory response-related genes and a downregulation of mRNA levels of innate immune-related genes in 3D4/21 cells. Treatment with CHR-6494 was found to reverse these transcriptional alterations, indicating that CHR-6494 mitigates PRV replication by regulating inflammation and innate immunity-related signaling pathways.

It is essential to recognize that the function of Haspin in relation to PRV infection has not been comprehensively examined in the current study. Firstly, the antiviral effectiveness of CHR-6494 against PRV infection has yet to be assessed *in vivo*. Secondly, the precise mechanism by which Haspin is implicated in PRV infection requires additional research.

## 5 Conclusion

In summary, this study represents the inaugural examination of the role of the *Haspin* gene in PRV infection and reveals the antiviral efficacy of CHR-6494 against both PRV and HSV-1 infections. The findings of this study enhance our understanding of the dynamics between PRV and its host, and present a promising molecular target for the future development of treatments for Herpesvirus infections.

## Data Availability

The original contributions presented in the study are included in the article/Supplementary material, further inquiries can be directed to the corresponding authors.

## Supplementary material

SUPPLEMENTARY FIGURE 1 CHR-6494 (200 nM) decreased the mRNA levels of PRV genes **(A)**, downregulated the mRNA levels of PRV-activated cytokine genes **(B)**, and upregulated the mRNA levels of PRV-suppressed innate immunity-related genes **(C)**.

SUPPLEMENTARY TABLE 1 Primer sequences used in this study.
