# Supplementary figures and images for: Haspin kinase inhibition dampens pseudorabies virus infection in vitro
Source: Front Vet Sci. 2025 Apr 23;12:1572729. doi: 10.3389/fvets.2025.1572729 (PMC12055825; doi:10.3389/fvets.2025.1572729)

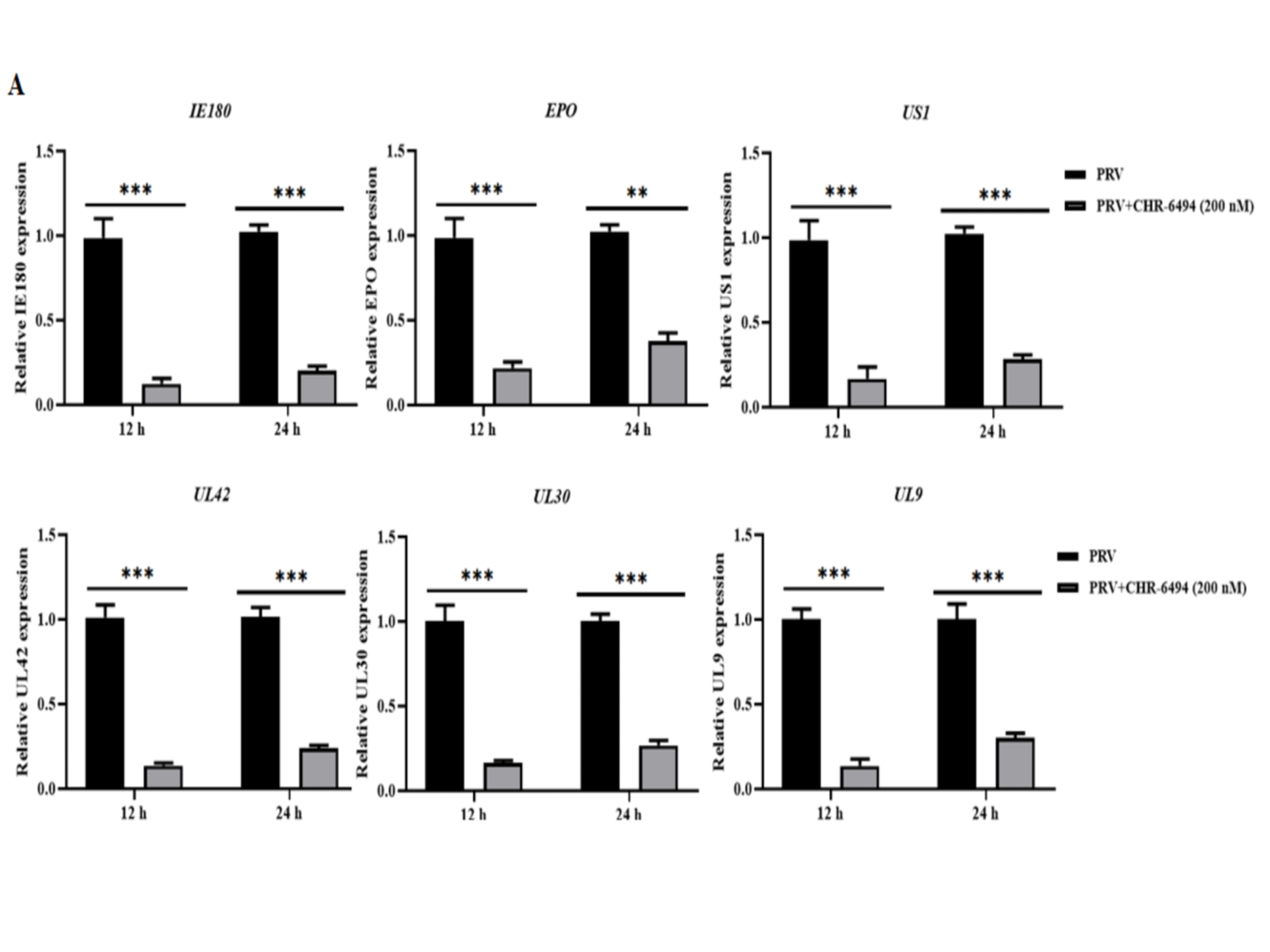

Supplement: SUPPLEMENTARY FIGURE 1 — CHR-6494 (200 nM) decreased the mRNA levels of PRV genes (A), downregulated the mRNA levels of PRV-activated cytokine genes (B), and upregulated the mRNA levels of PRV-suppressed innate immunity-related genes (C). [file Data_Sheet_1.zip › 新建文件夹/Supplementary Figure 1A.jpg]

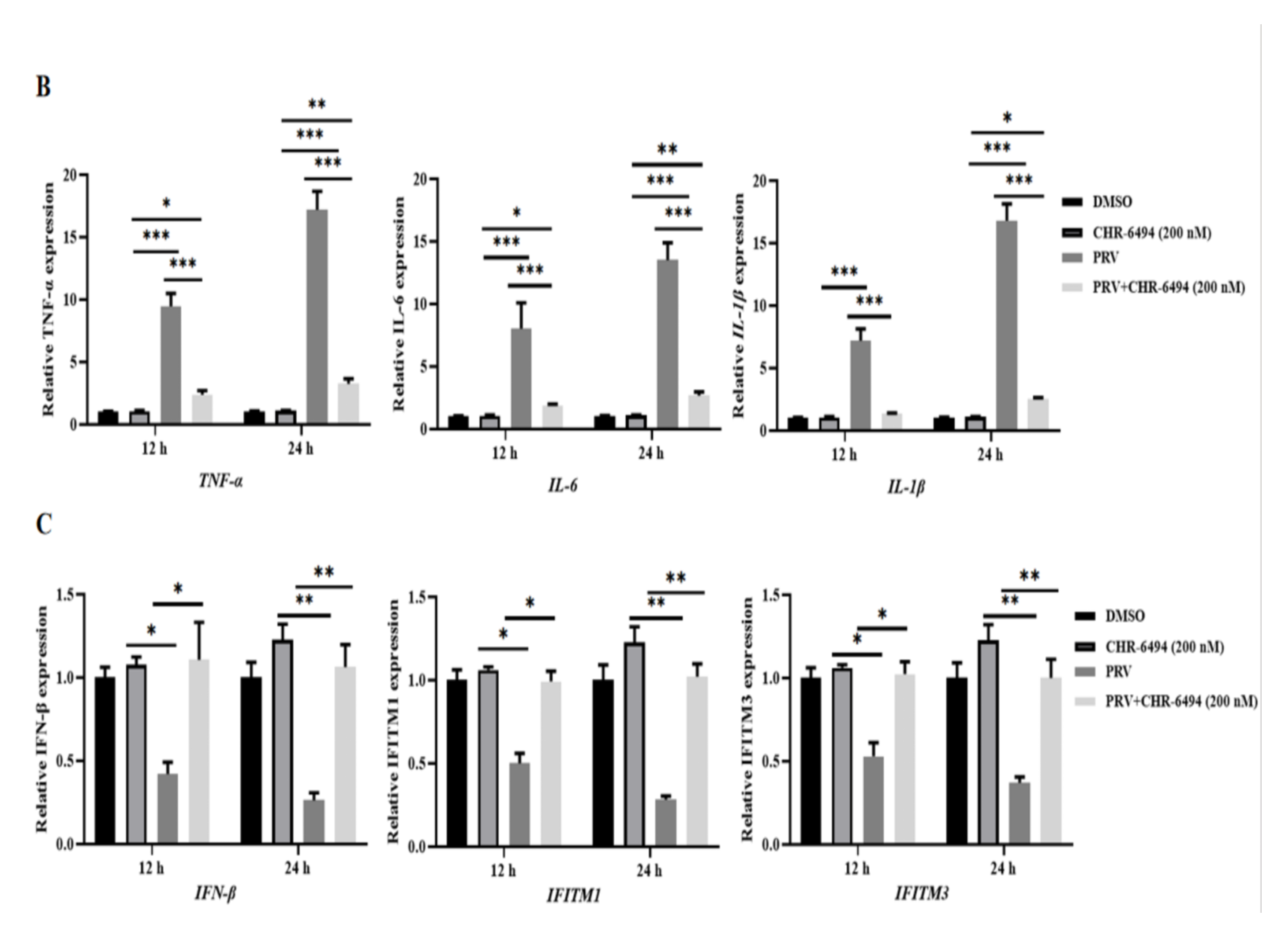

Supplement: SUPPLEMENTARY FIGURE 1 — CHR-6494 (200 nM) decreased the mRNA levels of PRV genes (A), downregulated the mRNA levels of PRV-activated cytokine genes (B), and upregulated the mRNA levels of PRV-suppressed innate immunity-related genes (C). [file Data_Sheet_1.zip › 新建文件夹/supplementary Figure 1B-C.jpg]
