# Supplementary material for: Haspin kinase inhibition dampens pseudorabies virus infection in vitro
Source: Front Vet Sci. 2025 Apr 23;12:1572729. doi: 10.3389/fvets.2025.1572729 (PMC12055825; doi:10.3389/fvets.2025.1572729)
Supplement: SUPPLEMENTARY TABLE 1 — Primer sequences used in this study. [file Table_1.docx]

**Supplementary Table 1:** Primer sequences used in this study.

| Gene | Sequence (3'-5') | Purpose |
| --- | --- | --- |
| Pig-GSG2 | F: AAGCCTGGGATCGCTACAAC;  R: TCGATCCCGCCAAACTCAAA | RT-qPCR |
| Mouse-GSG2 | F: CAGGCCTAAGAGAACTGGGC;  R: GGTCCAAGAATCCCTCTGGC | RT-qPCR |
| PRV-IE180 | F: CATCGTGCTGGACACCATCGAG;  R: ACGTAGACGTGGTAGTCCCCCA | RT-qPCR |
| PRV-EPO | F: GGGTGTGAACTATATCGACACGTC;  R: TCAGAGTCAGAGTGTGCCTCG | RT-qPCR |
| PRV-US1 | F: AGCTCAACGAGCGCGACGTCTA;  R: CGGAAGCTAAACTCGGACGCGA | RT-qPCR |
| PRV-UL42 | F: GCTCCCCGAGCGTCG;  R: CATGATGCAGTAGTCGTTGAACTC | RT-qPCR |
| PRV-UL30 | F: TCATCACGAAGAAGAAGTACATCGG | RT-qPCR |
|  | R: CCTTCATGAGCATCTTGCCG |  |
| PRV-gB | F: AAGTTCAAGGCCCACATCTA | RT-qPCR |
|  | R: TGAAGCGGTTCGTGATGG |  |
| PRV-gE | F: GACCCCGAGGACGAGTTCA | RT-qPCR |
|  | R: ACGCCATAGTTGGGTCCATT |  |
| PRV-UL9 | F: CAAGTTCAAGCACCTGTTCGA | RT-qPCR |
|  | R: TGAGGCTGTCGTTGACGC |  |
| HSV-1-TK | F: GCAGCAAGAAGCCACGGAAGT | RT-qPCR |
|  | R: AACCCAGGGCCACCAGCAGT |  |
| HSV-1-ICP27 | F: CGCCAAGAAAATTTCATCGAG | RT-qPCR |
|  | R: ACATCTTGCACCACGCCAG |  |
| Pig-NF-κB | F: ATGTCTGCACTTAGCCTCGATC  R: GAGTGCTTGGGCGGCCCCAG | RT-qPCR |
| Pig-TNF-α | F: CGACTCAGTGCCGAGATCAA  R: CCTGCCCAGATTCAGCAAAG | RT-qPCR |
| Pig-IL-6 | F: TGGATAAGCTGCAGTCACAG  R: ATTATCCGAATGGCCCTCAG | RT-qPCR |
| Pig-IL-1β | F: GCCCTGTACCCCAACTGGTA  R: CCAGGAAGACGGGCTTTTG | RT-qPCR |
| Pig-IFN-β | F: AGTTGCCTGGGACTCCTCAA | RT-qPCR |
|  | R: CCTCAGGGACCTCAAAGTTCAT |  |
| Pig-IFITM1 | F: CTGGGCTTCGTGGCTTTC |  |
|  | R: AACAGTGGCTCCGATGGTC |  |
| Pig-IFITM3 | F: GAATTGCGCTTCCCAGCCCTTCTT |  |
|  | R: GGAGGTCTCGCTTCGGATGTTGAT |  |
| Pig-GAPDH | F: ACCGTGTCTGCGACCTGA  R：ACCGTGTCTGCGACCTGA | RT-qPCR |
| Mouse-GAPDH | F: TGCTGGTGCTGAGTAGTGGTG | RT-qPCR |
|  | R:TCTTCTGGGTGGCAGTGATGG |  |
| pGSG2-shRNA-39 | F:CCGGGTCAGCTCTTCCGAACGTATGTCAAGAGCATACGTTCGGAAGAGCTGACTTTTT | Plasmid construction |
|  | R: AATTAAAAAGTCAGCTCTTCCGAACGTATGCTCTTGACATACGTTCGGAAGAGCTGAC |  |
| pGSG2-shRNA-668 | F: CCGGGCCTCAGGTCAGCTCTCTTTATCAAGAGTAAAGAGAGCTGACCTGAGGCTTTTT | Plasmid construction |
|  | R:AATTAAAAAGCCTCAGGTCAGCTCTCTTTACTCTTGATAAAGAGAGCTGACCTGAGGC |  |
| Pig-GSG2 | F: CGGCTAGCATGTACCCATACGACGTCCCAGACTACGCTATGGCGGCGTCACTCC | Plasmid construction |
|  | R: CGGAATTCTCACTTAAAGAGACTGTGC |  |
